# Field Programmable Gate Array Based Torque Predictive Control for Permanent Magnet Servo Motors

**DOI:** 10.3390/mi13071055

**Published:** 2022-06-30

**Authors:** Zheng Sun, Yikun Xu, Zhipeng Ma, Jun Xu, Tao Zhang, Muxun Xu, Xuesong Mei

**Affiliations:** 1 State Key Laboratory for Manufacturing Systems Engineering, Xi’an Jiaotong University, Xi’an 710049, China; zheng.sun@xjtu.edu.cn (Z.S.); disagreements@stu.xjtu.edu.cn (Y.X.); mazhipeng1016@stu.xjtu.edu.cn (Z.M.); xumuxun@mail.xjtu.edu.cn (M.X.); xsmei@xjtu.edu.cn (X.M.); 2 School of Electrical Engineering, Dalian University of Technology, Dalian 116024, China; zhangtaodlut@mail.dlut.edu.cn

**Keywords:** PMSM, FPGA, torque predictive control, motor drive

## Abstract

With the increasing demand for legged robots, the importance of the joint drive is increasing. The dynamic performance of the inner-most torque/current control loop conditions the capabilities of the whole joint system. In this paper, a direct torque control based on a prediction model is proposed. The motor torque is estimated by considering calculation and measurement delay; error estimation and torque tracking error are observed and compensated. The control algorithm was implemented on a Field Programmable Gate Array (FPGA) board to apply the capabilities of concurrency calculation of the FPGA. The effectiveness of the proposed control algorithm was experimentally verified. Compared with the commonly used Field Oriented Control (FOC) current controller, the presented controller can not only improve the dynamic performance of the motor but also reduce the average switching times of the inverter.

## 1. Introduction

The legged robot is an important branch of the service robot, configured to mimic the movement of living things in nature. In addition, as a kind of mobile robot, the legged robot can be used for dangerous or difficult tasks which are not suitable for humans, such as planetary exploration, disaster recovery operations, anti-terrorism, etc. [1]. Consequently, the issues of legged robots, including mechanical structure, stability analysis, control and drive algorithms have become an important research direction in the field of robotics in recent years [2]. With the increasing requirement for dynamic performance, the joint drive of the legged robot becomes more and more important. The highly customized robot joints consist normally of retarders and permanent magnet servo motors (PMSM), whose power density is much higher than that of induction motors. Traditionally, the joints are controlled by the cascade control structure with position, speed and current/torque control loops. With the increasing demand for environmental adaptability, impedance control can also be seen nowadays. Independently of which control strategy is applied, the dynamic performance of the innermost current or torque control loop conditions the capabilities of the whole joint system [3].

**Figure 1:**
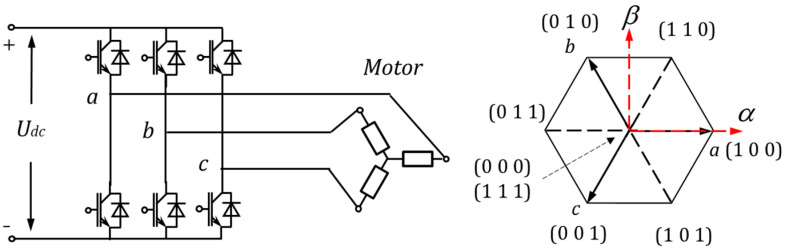
Three-phase inverter (**left**) and candidate voltage vectors (**right**).

The solution for driving a PMSM can be mainly divided into two categories, the current control and the torque control. The space vector pulse width modulation (SVPWM) is commonly used for the current control, where the reference voltage is synthesized by two neighboring candidate voltage vectors and a zero vector (see Figure 1) with a certain duty cycle calculated by the modulator. The motor current, as well as the torque generated by the SVPWM, is smooth, but the switching frequency for the inverters is constant, normally with a high value (16–32 kHz), even if the current reference is zero. It consequently results in high energy consumption and a low lifetime for the inverters [4]. Different from the current control, the direct torque control (DTC) estimates the flux linkage from the conducted voltage and chooses a certain candidate voltage through a lookup table for the desired torque change. Compared with the current control, the motor controlled by the DTC has a better tracking performance, but the torque ripple is also larger. Therefore, DTC is normally considered to be unsuitable for the precise servo system.

During the past decades, the finite-control model predictive torque control (FCS-MPTC) method has been regarded as an alternative method to DTC [5,6], which has been widely used in the electrical field [7,8]. FCS-MPTC utilizes the inherent discrete characteristics of modulatorless power inverters to solve optimization problems [5]. It inherits the fast response features of DTC while achieving other control goals, such as low switching frequency, energy consumption and current protection by multi-step prediction and cost function construction. Due to the limitations of fixed amplitude and phase angle of the candidate voltage, the torque ripple will be large when the control frequency is low [7].

In order to reduce torque ripple, many solutions have been proposed. Some scholars have found that two vectors can be used together in a single control cycle, usually to add the zero vector after the early optimization of the voltage vector [9,10,11]. To further predict the optimal situation, the duty cycle corresponding to each alternative vector can be calculated separately [12,13]. In addition, some scholars put forward the strategy of changing the switching point [14,15]. It is also effective to introduce virtual vectors to increase the number of alternative vectors [16,17,18,19], which could improve the accuracy of electromagnetic torque and stator flux. Using more complex electrical topologies and inverters to generate more alternative vectors is another option [20,21,22,23].

In essence, whether considering the duty ratio method or the virtual voltage vector expansion method, its core is still to reduce torque ripple by increasing the amount of calculation. Therefore, with the premise of keeping the switching frequency relatively low, a better control effect could be achieved by high-frequency calculation and reasonable cost function construction [24]. In this paper, a novel predictive torque control method to drive the PMSMs is proposed. Its principle is introduced in Section 2. A Field Programmable Gate Array (FPGA) is applied to implement the control algorithm, which is introduced in Section 3. With the capability of parallel computing of the FPGA, the prediction and control period can be reduced to 10^−5^~10^−6^ s, and achieve a better control performance. In Section 4, the effectiveness of the presented method is experimentally verified and analyzed. Section 5 concludes with comments.

## 2. Principle of Predictive Torque Tracking Control

**Figure 2:**
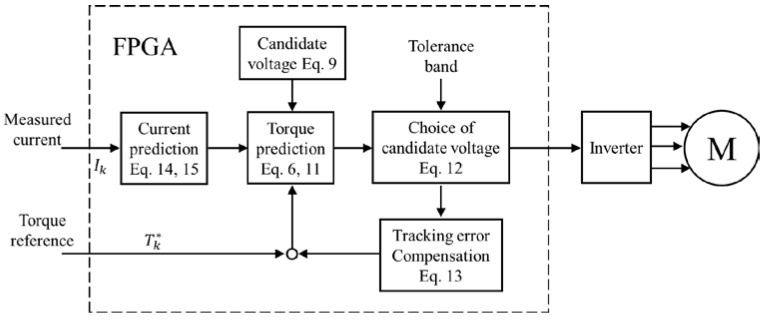
Schematic diagram of the proposed controller.

The proposed control structure is depicted in Figure 2, which combines current and torque prediction, tracking error accumulation, model error estimation and compensation. In the following text, the proposed model predictive direct torque control will be abbreviated as MPDTC. In this paper, the value with star denotes the reference value and the value with hut denotes the predictive value.

### 2.1. Prediction of the Motor Torque

For a 3-phase PMSM, the voltage equilibrium equation of a PMSM is always described in the dq-coordinate frame, which rotates with the rotor.

(1) $$\begin{array}{c} u_{d}=R_{d}i_{d}+L_{d}\frac{d}{dt}i_{d}-\omega_{e}L_{d}i_{q}-\psi_{q}\omega_{e}\\ u_{q}=R_{q}i_{q}+L_{q}\frac{d}{dt}i_{q}+\omega_{e}L_{q}i_{d}+\psi_{d}\omega_{e} \end{array}$$

where *ω_e_* is the electric rotational speed, $R=R_{d}=R_{q}=1.5\cdot R_{phase}$ is the resistance. *L* is the inductance, For the surface-mounted PMSM $L=L_{d}=L_{q}=1.5\cdot L_{phase}$. *Ψ* is the flux linkage of the permanent magnetic rotor, normally *Ψ_d_* = *Ψ_PM_*, *Ψ_q_* = 0. Equation (1) can be presented in matrix form by setting the state vector $I^{T}=\left[\begin{array}{cc} i_{d} & i_{q} \end{array}\right]$.

(2) $$\begin{array}{l} \left[\begin{array}{c} {\dot{i}}_{d}\\ {\dot{i}}_{q} \end{array}\right]=\left[\begin{array}{cc} -\frac{R}{L_{d}} & \omega_{e}\\ -\omega_{e} & -\frac{R}{L_{q}} \end{array}\right]\left[\begin{array}{c} i_{d}\\ i_{q} \end{array}\right]+\left[\begin{array}{cc} \frac{1}{L_{d}} & 0\\ 0 & \frac{1}{L_{q}} \end{array}\right]\left[\begin{array}{c} u_{d}\\ u_{q} \end{array}\right]+\left[\begin{array}{cc} 0 & \frac{\omega_{e}}{L_{d}}\\ -\frac{\omega_{e}}{L_{q}} & 0 \end{array}\right]\left[\begin{array}{c} \Psi_{PM}\\ 0 \end{array}\right]\\ \mathrm{or}\;\dot{I}=A_{c}\cdot I+B_{c}\cdot U+W_{c}\cdot \Psi \end{array}$$

Modern servo drivers are discrete controlled, so Equation (2) should be discretized by solving the equation with the current sampling period *T_s_*.

(3) $$\begin{array}{l} I_{k+1}=A_{d}\cdot I_{k}+B\cdot U_{k}+W\cdot \Psi \\ \mathrm{with}\;A_{d}=e^{A_{c}T_{s}}\;\;B=\int_{0}^{T_{s}}e^{A_{c}t}dt\cdot B_{c}\;\;W=\int_{0}^{T_{s}}e^{A_{c}t}dt\cdot W_{c} \end{array}$$

Since the magnitude order of $T_{s}$ is less than 10^−5^ s, the terms with more than one order of $T_{s}$ can be ignored. With the first-order Taylor expansion of $e^{A_{c}T_{s}}$, the discrete state equation can be approximated as follows.

(4) $$\begin{array}{l} \left[\begin{array}{c} i_{d,\;k+1}\\ i_{q,\;k+1} \end{array}\right]=\left[\begin{array}{cc} 1-\frac{RT_{s}}{L_{d}} & \omega_{e}T_{s}\\ -\omega_{e}T_{s} & 1-\frac{RT_{s}}{L_{q}} \end{array}\right]\left[\begin{array}{c} i_{d,\;k}\\ i_{q,\;k} \end{array}\right]+\left[\begin{array}{cc} \frac{T_{s}}{L_{d}} & 0\\ 0 & \frac{T_{s}}{L_{q}} \end{array}\right]\left[\begin{array}{c} u_{d,\;k}\\ u_{q,\;k} \end{array}\right]+\left[\begin{array}{cc} 0 & \frac{\omega_{e}T_{s}}{L_{d}}\\ -\frac{\omega_{e}T_{s}}{L_{q}} & 0 \end{array}\right]\left[\begin{array}{c} \Psi_{PM}\\ 0 \end{array}\right]\\ \mathrm{or}\;I_{k+1}=A_{d}\cdot I_{k}+B\cdot U_{k}+W\cdot \Psi \end{array}$$

To simplify the derivation in the following sections, Equation (4) will be reformed by introducing a new voltage vector *V*.

(5) $$\begin{array}{l} \left[\begin{array}{c} i_{d,\;k+1}\\ i_{q,\;k+1} \end{array}\right]=\left[\begin{array}{cc} 1-\frac{RT_{s}}{L_{d}} & 0\\ 0 & 1-\frac{RT_{s}}{L_{q}} \end{array}\right]\left[\begin{array}{c} i_{d,\;k}\\ i_{q,\;k} \end{array}\right]+\left[\begin{array}{cc} \frac{T_{s}}{L_{d}} & 0\\ 0 & \frac{T_{s}}{L_{q}} \end{array}\right]\left[\begin{array}{c} v_{d,\;k}\\ v_{q,\;k} \end{array}\right]\\ \mathrm{or}\;I_{k+1}=A\cdot I_{k}+B\cdot V_{k}\; \end{array}$$

(6) $$\mathrm{with}\;V_{k}=\left[\begin{array}{c} v_{d,\;k}\\ v_{q,\;k} \end{array}\right]=\left[\begin{array}{c} u_{d,\;k}+\omega_{e,\;k}L_{d}i_{q,\;k}\\ u_{q,\;k}-\omega_{e,\;k}L_{q}i_{d,\;k}-\psi_{PM}\omega_{e,\;k} \end{array}\right]$$

On the other hand, the motor flux linkage in the dq-coordinate frame can be summarized as

(7) $$\begin{array}{l} \Psi_{d}=L_{d}i_{d}+\Psi_{PM}\\ \Psi_{q}=L_{q}i_{q} \end{array}$$

Therefore, the torque generated by a 3-phase motor can be formulated as the function of the current, where *P* is the pole pair.

(8) $$T_{k}=\frac{3P}{2}\Psi_{k}\times I_{k}=\frac{3P}{2}\left[\Psi_{PM}i_{q,k}+\left(L_{d}-L_{q}\right)i_{d,k}i_{q,k}\right]$$

### 2.2. Choice of the Voltage Vector Considering One Step Delay

With a three-phase inverter, 7 different candidate voltage vectors can be generated, see Figure 1. Set a vector *S* = (a, b, c) to describe the status of the inverter, where a/b/c = 1 means the upper bridge is switched on, the lower bridge is switched off, and a/b/c = 0 means the upper bridge is switched off, the lower bridge is switched on. The vector *S* = (0, 0, 0) and *S* = (1, 1, 1) define the same status that the three phases of the motor are on equal potential. The remaining 6 statuses reform the DC bus voltage in 6 different directions as shown in Figure 1 right. These candidate vectors are described in the static orthogonal coordinate frame (αβ frame), whose α-axis matches the direction of phase a. According to the electrical angle *θ* from the α-axis to the d-axis, the candidate voltage vectors can be transformed in the dq-coordinate. Thus, the $u_{d}$ and $u_{q}$ in Equation (6) are determined.

(9) $$\begin{array}{l} U_{dq}=\left(\begin{array}{c} u_{d}\\ u_{q} \end{array}\right)=\left(\begin{array}{cc} \cos\theta & -\sin\theta \\ \sin\theta & \cos\theta \end{array}\right)\left(\begin{array}{c} u_{\alpha }\\ u_{\beta } \end{array}\right)\;\mathrm{with}\\ \left(\begin{array}{c} u_{\alpha }\\ u_{\beta } \end{array}\right)\in \left\{\begin{array}{lllllll} \left(\begin{array}{c} 1\\ 0 \end{array}\right) & \left(\begin{array}{c} \frac{1}{2}\\ \frac{\sqrt{3}}{2} \end{array}\right) & \left(\begin{array}{c} -\frac{1}{2}\\ \frac{\sqrt{3}}{2} \end{array}\right) & \left(\begin{array}{c} -1\\ 0 \end{array}\right) & \left(\begin{array}{c} -\frac{1}{2}\\ -\frac{\sqrt{3}}{2} \end{array}\right) & \left(\begin{array}{c} \frac{1}{2}\\ -\frac{\sqrt{3}}{2} \end{array}\right) & \left(\begin{array}{c} 0\\ 0 \end{array}\right) \end{array}\right\}\cdot U_{dc} \end{array}$$

**Figure 3:**
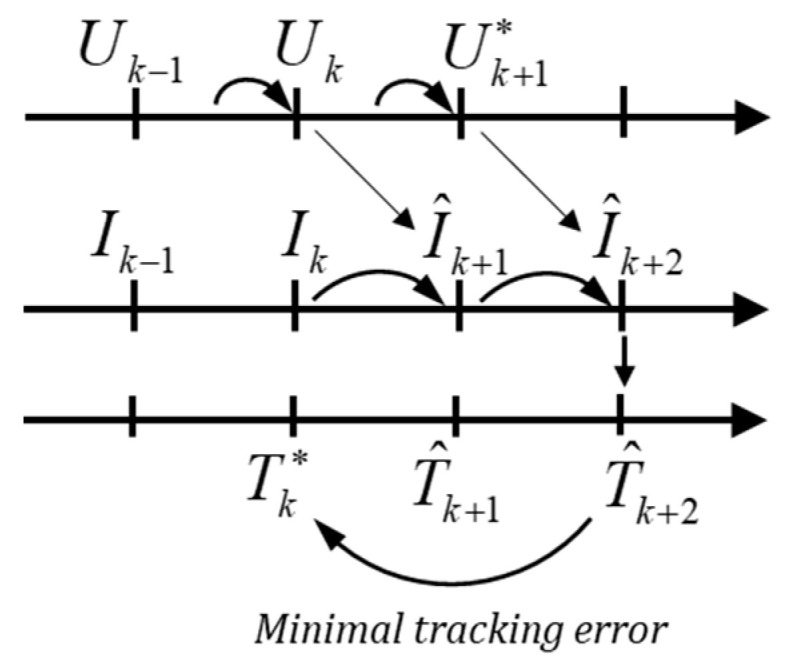
Time flow of the control process.

Figure 3 shows the time flow of the torque control. At moment *k*, the measured current $I_{k}$ will be changed to ${\hat{I}}_{k+1}$ under the action of $V_{k}$ (as well as $U_{k}$) generated between the *k* − 1 and *k* moment.

(10) $${\hat{I}}_{k+1}=A\cdot I_{k}+B\cdot V_{k}$$

The task of the controller is to find out the most suitable voltage for the next moment $U_{k+1}^{*}$ from 7 candidate voltage vectors to minimize the tracking error between the reference Torque received at this moment $T_{k}^{*}$ and predictive torque ${\hat{T}}_{k+2}$.

(11) $$\begin{array}{l} {\hat{I}}_{k+2}=A\cdot {\hat{I}}_{k+1}+B\cdot V_{k+1}^{*}\\ {\hat{T}}_{k+2}=\frac{3p}{2}\left[\Psi_{PM}{\hat{i}}_{q,k+2}+\left(L_{d}-L_{q}\right){\hat{i}}_{d,k+2}{\hat{i}}_{q,k+2}\right] \end{array}$$

Compared with the electromagnetic system, the mechanical response of a motor is much slower, so the rotational speed can be regarded as a constant value in the calculation, ${\hat{\omega }}_{k+1}=\omega_{k}$.

**Figure 4:**
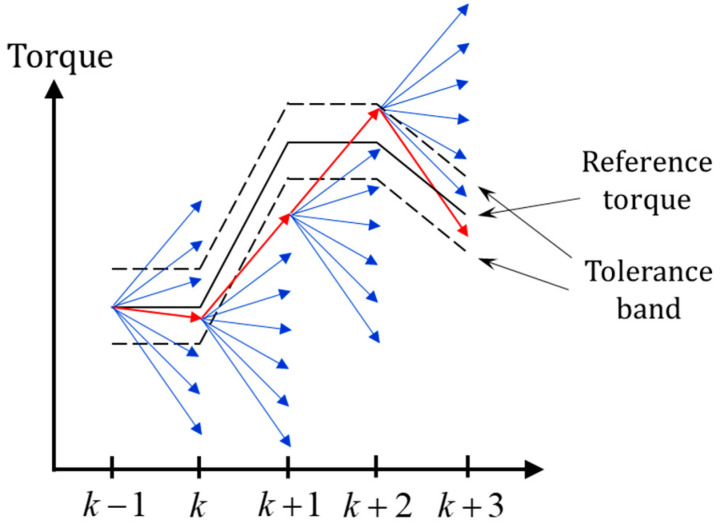
Choice of candidate vectors, blue: torque generated by the candidate voltage vectors, red: torque generated by the chosen vector.

A tolerance band is introduced to balance the torque tracking accuracy and other performance, as seen in Figure 4. If the change of the reference torque is too large that no candidate vector can generate the torque in the band, such as the situation from *k* to *k* + 1, the one with the minimal tracking error will be conducted at the next moment. If there are more candidates which enforce the torque in the band, such as the situation from *k* + 1 to *k* + 2, the one with the minimal value of the cost function will be conducted.

Since the torque accuracy is guaranteed through the tolerance band, the design of the cost function is considered with the aspect of the switching times and the current. At moment *k*, a certain voltage is conducted, and the original weight of the switching times from moment *k* to *k* + 1 can be found in Table 1, where the original weight is the switching times power of 2 to avoid the value 0 multiplied in the cost function Equation (12).

The cost function is designed as the multiplication of the weight of switching times and the motor predictive current,

(12) $$J=w_{s}^{p}\cdot \left|{\hat{I}}_{s,k+2}\right|$$

where ${\hat{I}}_{S,k+2}$ is the predictive current at moment *k* + 2 with the certain candidate voltage S, $w_{S}$ is the element from Table 1 and the index factor *p* is applied for adapting the weight between the switching times and the current.

### 2.3. Compensation for the Torque Tracking Error

As a side effect, the introduction of the tolerance band will lead to a static tracking error. In order to attenuate the static error, the torque reference will be modified with the tracking error from the previous step.

(13) $$\begin{array}{l} {\dot{T}}_{k}=K\left(T_{k-1}^{*}-{\hat{T}}_{k-1}\right)\\ T_{k}^{*}=T_{k,origin}^{*}+{\dot{T}}_{k}\cdot T_{s} \end{array}$$

where *K* is the gain to be turned and $T_{s}$ is the control period. The effect of such a modification can be regarded as an integrator, which accumulates the previous tracking error and compensates it into the reference value with a certain factor.

Besides the static error, the model used for the prediction (Equations (10) and (11)) is not free from deficiencies, since the motor resistance may be changed by the temperature, the switching should have dead time to avoid short circuits, etc. To improve the prediction accuracy, an observer is applied to estimate and compensate for the prediction error. Since the matrices *A* and *B* in Equation (10) are diagonal matrices, the observer can be designed independently. For d or q-phase, Equation (10) can be simplified as a scalar equation.

(14) $${\hat{i}}_{k+1}=i_{k}+\frac{RT_{s}}{L}i_{k}+\frac{T_{s}}{L}v_{k}+\frac{T_{s}}{L}\varepsilon_{k}$$

where *ε* denotes the lumped model error, which can be detected through the comparison of measured and estimated current.

(15) $$\varepsilon_{k}=K_{p}\left(1+K_{i}\frac{T_{s}}{z-1}\right)\left(i_{k}-{\hat{i}}_{k}\right)$$

where $K_{p}$ and $K_{i}$ are two parameters to be tuned. Insert Equation (14) into Equation (13) and substitute ${\hat{i}}_{k+1}$ with $z{\hat{i}}_{k}$ and the following equation can be obtained.

(16) $$\left[z+\frac{T_{s}}{L}\left(K_{p}+\frac{K_{p}K_{i}T_{s}}{z-1}\right)\right]{\hat{i}}_{k}=\left[1+\frac{RT_{s}}{L}+\frac{T_{s}}{L}\left(K_{p}+\frac{K_{p}K_{i}T_{s}}{z-1}\right)\right]i_{k}+\frac{T_{s}}{L}v_{k}$$

Therefore, the observer is stable only if the roots of the following equation locate in the unit circle.

## 3. Implementation in FPGA

As a kind of semi-custom circuit, FPGA can hardware the system into the actual electronic circuit through the compilation and synthesis of hardware description language (HDL), which can be prepared for the development of custom-specific driver control chips. Therefore, the FPGA is applied as the platform to implement the algorithm of MPDTC proposed in this paper.

**Figure 5:**
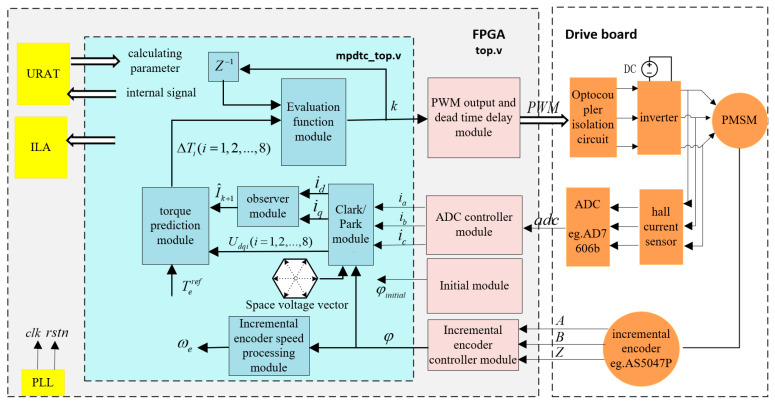
FPGA architecture design block diagram.

The design of FPGA adopts the top-down modular design method, including the top-level module, the core algorithm module, the peripheral interface control module and the upper computer receiving and transmitting module. Figure 5 shows the architecture diagram of the proposed FPGA drive system.

**Figure 6:**
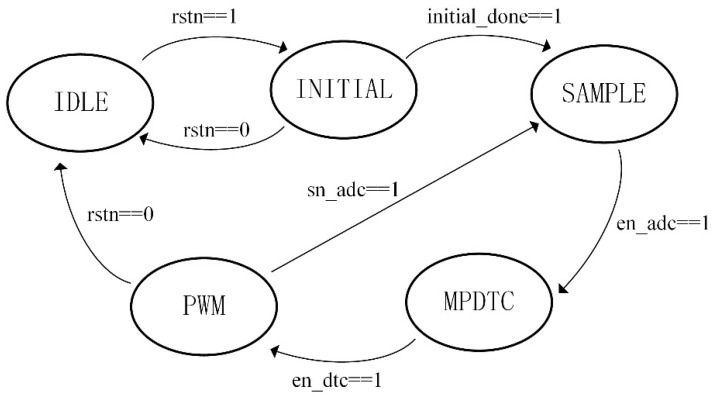
FSM status transfer diagram.

A finite synchronous state machine (FSM) is used for the timing planning of the FPGA system. According to the time sequence, the FPGA system can be divided into five stages: IDLE, INITIAL, SAMPLE, MPDTC and PWM. The FPGA system is in the IDLE state at the beginning of the work. After the system reset, the system clock of the FPGA system begins to work. Then, the system runs into the INITIAL state. The controller rotates the motor to the position where the d-axis matches the a-axis. Then, the system runs into the SAMPLE state to collect the current and encoder angle signal through the ADC and digital inputs, respectively. After the signal collection, it enters the MPDTC algorithm processing state to find out the most suitable candidate voltage. Finally, the FPGA system outputs the switching signal of the selected voltage to complete a control cycle. Figure 6 shows the transition of the FSM status.

## 4. Experimental Verification

**Figure 7:**
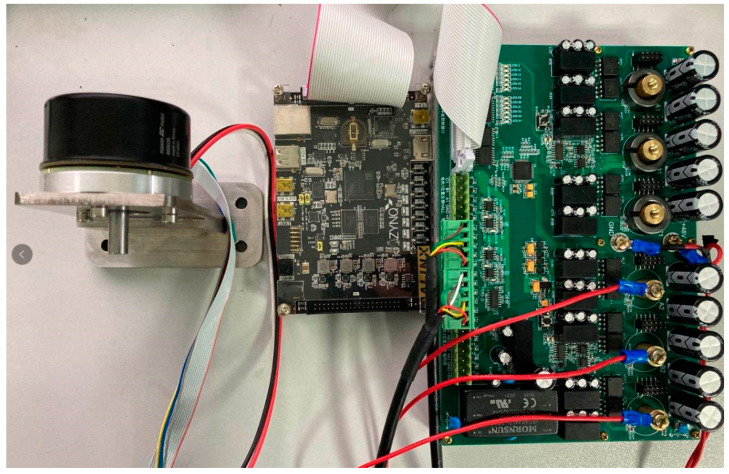
Test bench.

The test bench to verify the effectiveness of the proposed control method is depicted in Figure 7. The control algorithm is implemented in the ZynQ-7000 development board (ALINX Electronic Technology Co., Ltd., Shanghai, China) with a sampling frequency of 256 kHz and a control frequency of 64 kHz. Each four sampled phase current and motor angles are averaged for the controller to reduce the measurement noise. For comparison, a PI current controller with the SVPWM is also implemented in the development board with a control frequency of 16 kHz, which is abbreviated as FOC in the following.

The generated signals for the IGBT are sent to a self-made power board with a three-phase inverter. A high-dynamic synchronous motor EC60-647695 from Maxon (Maxon motor ag, Sachseln, Switzerland) is connected with the power board for the experiments. The parameters of the motor are listed in the Table 2.

**Figure 8:**
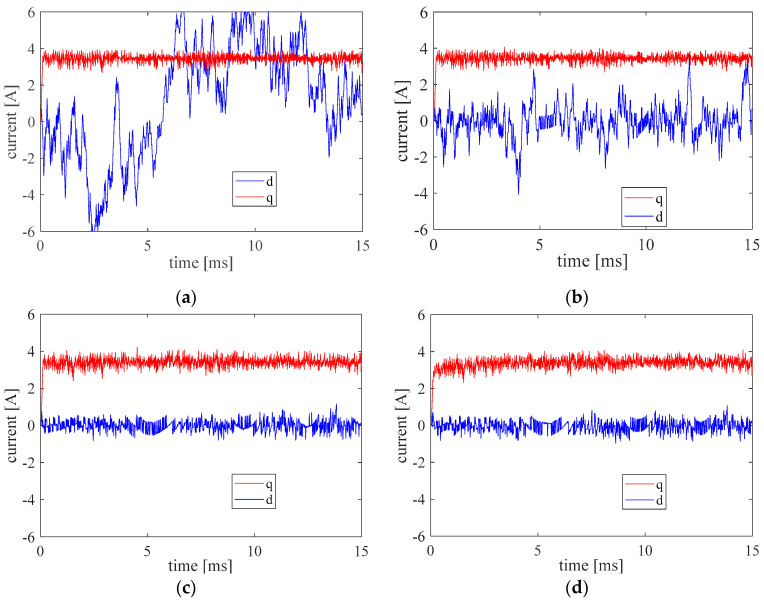
The current of d and q axes with different torque tolerance. (**a**) $T_{tol}=0.02\;\mathrm{Nm}$, (**b**) $T_{tol}=0.04\;\mathrm{Nm}$, (**c**) $T_{tol}=0.08\;\mathrm{Nm}$, (**d**) $T_{tol}=0.12\;\mathrm{Nm}$.

The influence of the tolerance band ($T_{tol}$) on the control performance was investigated first, and the results are plotted in Figure 8. In the test, the torque step of 0.4 Nm is applied at *t* = 0.1 ms. If the tolerance is too small, there is no candidate voltage located in the tolerance band, so the choice of the voltage only depends on the tracking error between the reference and the predictive torque. Consequently, it results in a large current value of the d-axis and a high switching frequency ($f_{sw}$). It can be seen from Table 3, that with the increasing $T_{tol}$, the current value of the d-axis is decreased. However, if $T_{tol}$ is too large, the cost function will choose the voltage with minimal switching times and current, so the dynamic performance is damaged.

The tolerance band in the following experiments was set with the value of 0.08 Nm. The influence of the switching time weighting *p* in Equation (12) is then tested. The reference torque was given as the same as the test for $T_{tol}$. The results are listed in Table 4. The value of p has less effect on the settling time of the current or the torque. However, it is obvious that the weighting parameter *p* has the effect of reducing the average switching frequency. As a side effect, the meaningless current (here the current of the d-axis) is also increased, since the large value of *p* means the decreased relative weighting of current in the cost function.

**Figure 9:**
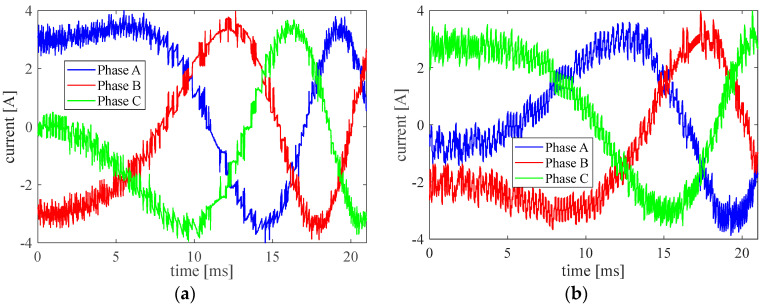
The current of three phases. (**a**) MPDTC, (**b**) FOC.

The parameter p=0.1 and $T_{tol}=0.08$ Nm is chosen for the following experiments. With these parameters, the current of the three phases is plotted in Figure 9a. For comparison, the phase current from the FOC is presented in Figure 9b. Because of the reduction of the unnecessary switching, the MPDTC shows a smoother phase current with less noise in some places.

**Figure 10:**
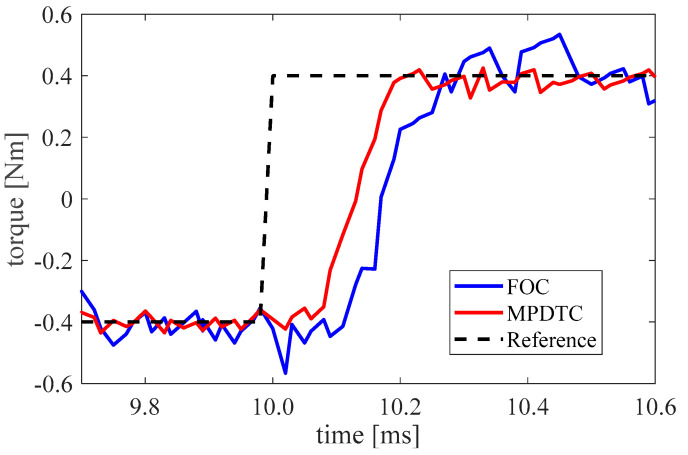
Comparison of the step response.

The step response of MPDTC and FOC is compared in Figure 10. For this test, the reference torque is switched to be −0.4 Nm and 0.4 Nm each 10 ms. The generated motor torque here is calculated offline through the measured phase current and the motor angle. Compared with the FOC, the presented MPDTC decreases the torque settling time by 30% from 0.27 ms to 0.19 ms. It means the proposed method shows a better dynamic response with fewer switching times than the commonly used FOC current control.

**Figure 11:**
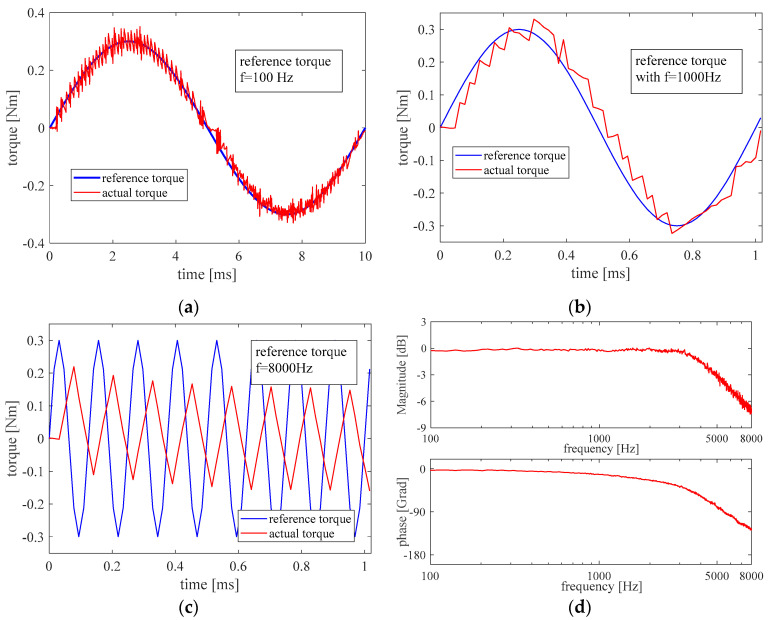
Sine tracking performance with the frequency of 100 Hz (**a**), 1000 Hz (**b**), and 8000 Hz (**c**), and the Bode plot of the closed torque control loop (**d**).

The benefits of dynamic performance can also be seen in the frequency domain. Figure 11 shows the tracking performance of the sine reference with different frequencies and the Bode-plot after the sine sweep. The amplitude of the reference torque is 0.3 Nm and the frequency is swept from 100 Hz to 8000 Hz. The generated torque is calculated through the measured motor current. It can be seen that the bandwidth of the torque close loop is about 5000 Hz, which also matches the result from the step response. The controlled motor can follow the sine torque reference well when the frequency is lower than the bandwidth.

## 5. Conclusions

A novel model predictive torque control method for PMSM is presented in this paper to meet the dynamic demand of legged robots. The motor torque is estimated by considering the delay of calculation and measurement. A tolerance band is introduced to balance the tracking accuracy and switching frequency. The estimating error and the accumulative torque tracking error are observed and compensated. The control algorithm was implemented on an FPGA board, where the torque generated by the 7 candidate voltages can be calculated in parallel. The influence of the value of the torque tolerance band and the weighting for switching times is experimentally analyzed. Compared with the commonly used FOC current controller, the proposed MPDTC can not only improve the dynamic performance of the motor but also reduce the average switching times of the inverter.

## Figures and Tables

**Table 1:** The original weight of the switching times.

	*S*_*k*+1_	000	001	010	011	100	101	110	111
*S_k_*									
000		1	2	2	4	2	4	4	8
001		2	1	4	2	4	2	8	4
010		2	4	1	2	4	8	2	4
011		4	2	2	1	8	4	4	2
100		2	4	4	8	1	2	2	4
101		4	2	8	4	2	1	4	2
110		4	8	2	4	2	4	1	2
111		8	4	4	2	4	2	2	1

**Table 2:** Parameters of the applied motor.

Description	Value	Unit
Terminal resistance	1.11	Ω
Terminal inductace	1.28	mH
Torque constant	113	mNm/A
Speed constant	84.8	rpm/V
Rotor inertia	810	gcm^2^
Nominal voltage	48	V

**Table 3:** Influence of $T_{tol}$ on the control performance.

$T_{tol}\;[\mathrm{Nm}]$	0.02	0.04	0.08	0.12
$\left|I_{d,max}\right|$ **[A]**	7.89	4.28	1.05	1.07
**Settling time of** $I_{q}$ **[ms]**	0.12	0.12	0.18	0.38

**Table 4:** Influence of p on the control performance.

p	0.02	0.1	0.15	0.2
$\left|I_{d,max}\right|$ **[A]**	1.07	1.06	2.02	4.13
**Average** $f_{sw}$ **[kHz]**	16.7	14.0	12.7	9.2

## Data Availability

Not applicable.
